# Long-term pain outcomes after serial lidocaine infusion in participants with recent onset of peripheral neuropathic pain: A pilot double-blind, randomized, placebo-controlled trial

**DOI:** 10.1097/MD.0000000000038253

**Published:** 2024-05-24

**Authors:** Suratsawadee Wangnamthip, Pramote Euasobhon, Kasamabhorn Thiangtham, Sukunya Jirachaipitak, Pranee Rushatamukayanunt, Mark P. Jensen

**Affiliations:** a Department of Anesthesiology, Faculty of Medicine, Siriraj Hospital, Mahidol University, Bangkok, Thailand; b Division of Anesthesia, Thungsong Hospital, Nakhon Si Thammarat, Thailand; c Department of Rehabilitation Medicine, University of Washington, Seattle, Washington, USA.

**Keywords:** adult, drug therapy, intravenous, lidocaine, neuropathic pain, pain management, randomized controlled trials

## Abstract

**Background:**

This study investigated the outcomes up to 12 weeks after serial lidocaine infusion for early-onset peripheral neuropathic pain.

**Methods:**

This pilot double-blind, randomized, 2-arm placebo-controlled trial recruited 50 participants with onset of peripheral neuropathic pain within the past 6 months and randomized them to either receive lidocaine (3 mg/kg) in normal saline (50 mL) intravenous infusion over 1 hour (lidocaine group) once a week for 4 weeks or 50 mL of normal saline infusion (placebo group) once a week for 4 weeks. Twenty-nine participants completed the protocol; 15 participants were assigned to the lidocaine group and 14 to the placebo group. The outcomes were pain intensity assessed using a numerical rating scale (NRS), quality of life assessed using EuroQol-Five Dimensions-Five Levels questionnaire (EQ-5D-5L), psychological function using the Thai version of the 21-item Depression Anxiety Stress Scales (DASS-21), pain medication use, and adverse effects, all assessed at baseline (BL) and again at 4, 8, and 12 weeks following randomization.

**Results:**

The reported tramadol use at 8 and 12 weeks following the first infusion was significantly lower in the lidocaine group (*P = *.023). No other significant between-group differences were observed at any time point or for any other outcome, and no serious adverse events were observed.

**Conclusion:**

Multiple lidocaine infusions of 3 mg/kg once a week for 4 weeks in participants with recent onset of peripheral neuropathic pain demonstrated no significant benefits in pain intensity, quality of life, or psychological outcomes. At most, this treatment may result in less tramadol use.

## 1. Introduction

Peripheral nerve diseases or lesions can cause severe and long-lasting pain. The prevalence of pain predominantly of peripheral neuropathic origin has been reported to be 7% to 10% in the general population.^[1,2]^ Unfortunately, the available treatments for neuropathic pain result in inadequate pain control.^[3]^ Moreover, severe and prolonged intractable pain can contribute to suffering and poor quality of life.^[4]^ Although the use of multiple medications at high doses may provide limited relief, side effects, including sedation, dizziness, nausea, vomiting, and constipation may be intolerable for many.^[5,6]^

Central sensitization is one of the mechanisms that can contribute to chronic neuropathic pain. The causes of central sensitization include increased activity from peripheral nerves that decreases the participant’s natural threshold, leading to an exaggerated response to suprathreshold stimuli, which can contribute to dispersion of pain beyond the injury site, wind-up-like phenomenon, and aftersensation.^[7]^ Effective early treatment could potentially prevent or minimize central sensitization and improve long-term outcomes.^[8]^

Lidocaine, a sodium channel blocker, is known to block spontaneous ectopic activity without suppressing normal nerve conduction.^[9]^ Lidocaine also has potent anti-inflammatory properties that are greater than conventional anti-inflammatory drugs.^[10]^ Moreover, lidocaine has been found to be associated with a reduction in circulating inflammatory cytokines, which plays a role in the process of secondary hyperalgesia and central sensitization.^[11]^ Lidocaine also provides other molecular mechanisms involving along the pain pathway, such as M1 and M3 muscarinic receptor blockage and NMDA receptor antagonist.^[12]^

Lidocaine can also relieve some acute and chronic pain conditions.^[13,14]^ Intravenous lidocaine has been used to treat refractory neuropathic pain since 1950.^[15]^ Later studies and meta-analyses have confirmed the efficacy of lidocaine infusions for reducing the intensity of a variety of neuropathic pain conditions, such as spinal injury pain, central pain syndromes, diabetic peripheral neuropathy, postherpetic neuralgia, trigeminal neuralgia, and complex regional pain syndrome.^[16,17]^ More recent studies have found intravenous lidocaine therapy to be effective for treating central and peripheral neuropathic pain.^[18]^

The total dose of lidocaine infusion used to treat neuropathic pain has commonly ranged from 1 to 5 mg/kg.^[16,19]^ The pain relief following a single lidocaine infusion normally lasts for only a few days but can occasionally last for several days or weeks.^[19]^ Although clinical trials have evaluated the short-term efficacy of multiple lidocaine infusions in various chronic pain conditions, no study has evaluated this intervention in early-onset of neuropathic pain and its longer-term efficacy of up to 12 weeks.^[17]^

Prior to the commencement of the current study, we considered a number of factors regarding the dose of lidocaine to study, the study population, and the best follow-up period. Following this discussion, we determined that the 3 mg dose is 1 that clinicians commonly use, so it would be important to evaluate its effects, the 3 mg has fewer side effects than larger doses, so if this dose was found to be effective, this would provide important information, an early-onset peripheral neuropathic pain population is important to study, given their risk for the development of persistent pain, and the possibility of reducing that risk with lidocaine treatment, and longer-term outcomes (e.g., up to 12 weeks) are rarely studied, yet findings regarding the longer-term outcomes (e.g., the development of chronic pain over the longer-term) are important.

We, therefore, conducted this prospective 2-arm double-blinded, randomized, placebo-controlled trial to evaluate primarily the potential short- and longer-term (i.e., 12 weeks after treatment started) benefits of serial lidocaine infusions 3 mg/kg provided once a week for 4 weeks in a sample of participants with early-onset peripheral neuropathic pain. Secondary objectives were to evaluate the effects of this treatment protocol on pain immediately after infusion, oral pain medication use, quality of life, pain-related psychological function, and any adverse events associated with the lidocaine infusion. Given the inconsistent findings in prior research regarding the efficacy of lidocaine infusions,^[16]^ as well as the lack of any studies that have examined the effects of lidocaine infusions in individuals with early-onset pain, we did not have a strong empirical basis for hypothesizing significant effects for the treatment or for determining the sample size needed to detect an effect. We therefore viewed this as a preliminary study, the findings from which could inform future research in this area.

## 2. Methods

### 2.1. Study design

This double-blind, 2-arm randomized, placebo-controlled trial included participants with peripheral neuropathic pain of onset within 6 months who were treated at the Pain Clinic, Department of Anesthesiology, Faculty of Medicine Siriraj Hospital, Mahidol University, Bangkok, Thailand. The protocol for this study was approved by the Siriraj Institutional Review Board (COA no. 140/2014). This study was registered at clinicaltrials.gov (reg. no. NCT02217267) prior to participant enrollment and complied with all principles set forth in the Declaration of Helsinki (1964) and all of its subsequent amendments. Written informed consent was obtained from all participants.

### 2.2. Participants

Individuals aged 18 to 75 years with a diagnosis of peripheral neuropathic pain of any etiology and a duration of less than 6 months were eligible. In order to participate, the potential participant had to be able to read, write, and understand the Thai language evaluation form. Neuropathic pain was defined as the presence of pain deemed to be associated with lesions or diseases of somatosensory nervous system and/or sensory abnormalities (burning, electrical shocks, tingling, pins and needles, and/or numbness in the affected area). Individuals with 1 or more of the following were excluded: history of cardiovascular disease with 12-lead electrocardiogram-proven conduction abnormalities; allergy to lidocaine, gabapentin, or tramadol; history of epilepsy; history of previous lidocaine infusion, psychiatric disease, or drug abuse; pregnancy and/or breastfeeding. Withdrawal or termination criteria developing serious adverse effects from lidocaine, gabapentin, carbamazepine, and/or tramadol, or being lost to follow-up. All participants were treated according to the standard of care protocol for this condition at our treatment center, including receiving full information regarding the expected benefits and possible complications of the study treatment.

### 2.3. Interventions

The lidocaine group received lidocaine (3 mg/kg) diluted in 50 mL of normal saline, and the placebo group received normal saline (50 mL), which was manufactured by pharmacists from the Siriraj Hospital Pharmacy Department. The solution was infused over a period of 1 hour, once a week for 4 weeks. During the 4 infusion sessions, participants were continuously monitored by blood pressure manometer, 3-lead electrocardiogram, and pulse oximetry. Systolic blood pressure, diastolic blood pressure, heart rate, and oxygen saturation were recorded every 15 minutes. Any adverse effects, such as arrhythmia, dizziness, tinnitus, perioral numbness, tongue paresthesia, and urticarial rash, were documented. Infusion was stopped when any side effect of concern was observed.

Numerical rating scale (NRS) pain measurement was initiated 15 minutes before drug infusion and again 15 minutes after completion of the infusion. Follow-up visits to evaluate the reduction of pain were scheduled for the end of week 8 and the end of week 12. EuroQol-Five Dimensions-Five Levels (EQ-5D-5L) and the Thai version of the 21-item Depression Anxiety Stress Scales (DASS-21) questionnaires were administered at week 1 before infusion and at the end of week 12.

All participants received an anticonvulsant (gabapentin) and weak opioids (tramadol) according to the following protocol. Gabapentin (300 mg) once daily was prescribed. If an NRS score of greater than 4 persisted, additional gabapentin (100–300 mg) could be given every 3 to 7 days, if tolerated. If an NRS score greater than 4 continued to persist and the participant reported pain-related insomnia, a tricyclic antidepressant (amitriptyline or nortriptyline, 10 mg) could be given before bedtime and titrated every 3 to 7 days if tolerated. For rescue pain medication, we prescribed 1 or 2 capsules of tramadol (50 mg) orally when necessary every 6 hours (maximum 8 capsules/day). Medications used, total mg/day of each medication were recorded at every visit and calculated to sum of each week from (1^st^-2^nd^ week, 2^nd^-3^rd^ week, 3^rd^-4^th^ week, 5^th^-8^th^ week, and 9^th^-12^th^ week), and adverse effects of medications were recorded at every visit.

### 2.4. Data collection and outcome measures

The primary outcome of this study was pain intensity assessed at week 12 following the initial infusion. Secondary outcomes were oral analgesic usage, quality of life, pain-related psychological effects, and adverse effect of lidocaine infusion. Data were collected at baseline (BL/Week 1) and at weeks 2, 3, 4, 8, and 12. BL demographic data (age, gender, weight, BMI, medical conditions) were recorded. The diagnosis of peripheral neuropathic pain, its location, intensity, and the Thai version of the Douleur Neuropathique 4 questionnaire (DN4)^[20]^ were also recorded at BL. Other variables such as blood pressure, heart rate, and electrocardiography during lidocaine/normal saline infusion were monitored. Average pain intensity pre- and postinfusion was also assessed 4 times during the intervention. The average pain intensity in the previous 7 days, analgesic use in the past week (including anticonvulsants [gabapentin, pregabalin, carbamazepine, and oxcarbazepine], antidepressants [tricyclic antidepressants], and weak opioids [tramadol] were recorded as average mg/day at every assessment point. Quality of life and pain-related psychological outcome domains were recorded at BL/week 1, and week 12 and adverse effects of drug were recorded during the infusion. The specific measures used to assess the study outcomes are presented below.

### 2.5. Pain intensity

A 0 to 10 NRS was used to assess average pain intensity in the previous 7 days at BL (i.e., week 1), week 2, week 3, week 4, week 8, and week 12. The NRS was also used to assess current pain intensity 15 minutes before and immediately after each of the 4 infusions. The NRS responses can range from 0 to 10 with “0” indicating “No pain,” and “10” indicating the “Worst pain imaginable.”^[21]^ The percentage of pain reduction was calculated by (Pretreatment NRS-Posttreatment NRS) *100/Pretreatment NRS for calculating immediate pain reduction after infusion or (BL/Week 1 NRS-Week 12 NRS)*100/BL/week 1 NRS for calculating the pain reduction at week 12.

### 2.6. Quality of life

A self-report Thai version of the EuroQol-Five Dimensions-Five Levels questionnaire (EQ-5D-5L) was used to assess quality of life.^[22,23]^ With this measure, respondents rate the extent to which they have problems in each quality of life domain assessed (i.e., mobility, self-care, usual activities, pain/discomfort, and anxiety/depression) using a 5-point Likert scale (No problems, Slight problem, Moderate problems, Severe problems, and Unable to/Extreme problem). In addition, the EQ-5D-5L includes a “utility score” item assessing overall quality of life can range from 0 (representing death) to 1 (representing full health). However, respondents can rate their health as being “worse than death,” by providing a score <0 if they wish. For example, in Thai populations, the score has been shown to range from −0.42 to 0.94.^[23]^ Participants were also asked to rate their overall health using a 100 mm Visual Analog Scale, with “Worst imaginable health” and “Best imaginable health” as the endpoints.

### 2.7. Psychological function

The Thai version of the DASS-21.^[24,25]^ was used to assess depression, anxiety, and stress. Each of these domains is assessed with a separate 7-item scale. Responses to the items are summed to create scales with a total possible score of 21 points. The final score can then be graded in each domain: Normal (0–4), Mild (5–6), Moderate (7–11), Severe (11–13) and Extremely Severe (14+) for depression; Normal (0–3), Mild (4–5), Moderate (6–7), Severe (8–9) and Extremely Severe (10+) for anxiety; and Normal (0–7), Mild (8–9), Moderate (10–12), Severe (13–16), and Extremely Severe (17+) for stress.

### 2.8. Adverse events

Possible adverse events sometimes associated with lidocaine infusion include dizziness, tinnitus, perioral numbness, tongue paresthesia, and urticarial rash. Their presence was assessed using a 3-point scale. A “0” meant the adverse event was “Not present,” a “1” meant that the adverse event was present, but did not require treatment, and a “2” meant that the adverse event was present, and required treatment. In addition, the presence or absence of cardiac arrhythmia during the infusions was monitored by nurses during the infusion.

### 2.9. Sample size calculation

As noted in the Introduction, no prior research has been conducted to study the effects of serial low-dose lidocaine infusions in individuals with early-onset neuropathic pain. We therefore did not have a basis for determining the sample size needed for a definitive test of the possible benefits of this treatment, and therefore chose to conduct this as a preliminary study. Based on prior research in the area that has used sample sizes ranging from 15^[26]^ to 21^[27]^ per condition, we planned to enroll over 30 participants (i.e., at least 15 subjects per condition). The sample size of this pilot study was 15 per group according to considerations in determining sample size for pilot studies suggesting a sample ranging in size from 10 to 40 per group, providing estimates precise enough to meet a variety of aims.^[28]^

### 2.10. Randomization and blinding

Participants’ group assignment was determined by computer-generated randomization using nQuery Adviser 6.0 software (Statistical Solutions, Inc., Cork, Ireland). Group assignments of individual participants were sealed in opaque envelopes to ensure that participants and physicians would be blinded to treatment allocation. The study group received 3 mg/kg of lidocaine in 50 mL normal saline, and the control group received 50 mL of normal saline. The infusion fluids were prepared by pharmacy staff so that the appearance and volume of both preparations were the same. Both groups had an infusion time of 1 hour. During the infusion, the investigator who administered the medications, participants, and the assessor were blinded to group assignment.

### 2.11. Statistical analysis

SPSS Statistics version 18 (SPSS, Inc., Chicago, IL) was used for all statistical analyses. The number and percent of the categorical descriptive variables, including gender, diagnosis, location of pain, and DASS-21 severity, were computed. The Shapiro–Wilk test with an alpha.05 level was used to evaluate normality. Medians and interquartile ranges were computed for the continuous descriptive data (age, weight, body mass index [BMI], 0–10 NRS, DN4 score, medication dose [mg/day], and EQ-5D-5L utility scores). The Mann–Whitney U test was used to compare reductions in pain intensity between groups. The effect size was calculated with the Mann–Whitney U test “r = Z/ √N.” Pearson Chi-square or Fisher exact test was used to compare categorical data between groups. Continuous outcome measures, including the 0-10 NRS and analgesic dose, were analyzed using Friedman test within each group (alpha < 0.01 was set as the significance level). post hoc analyses, using the Bonferroni method to control for multiple tests, were conducted to evaluate the direction of any differences found. Wilcoxon Sign Rank test was used to analyze paired measurements of continuous data and categorical data, respectively. Analysis of covariance was used to adjust the BL pain score in both groups. The linear-mixed model was applied to analyze repeated measures in longitudinal outcomes.

## 3. Results

### 3.1. Study sample description

**Figure 1.**
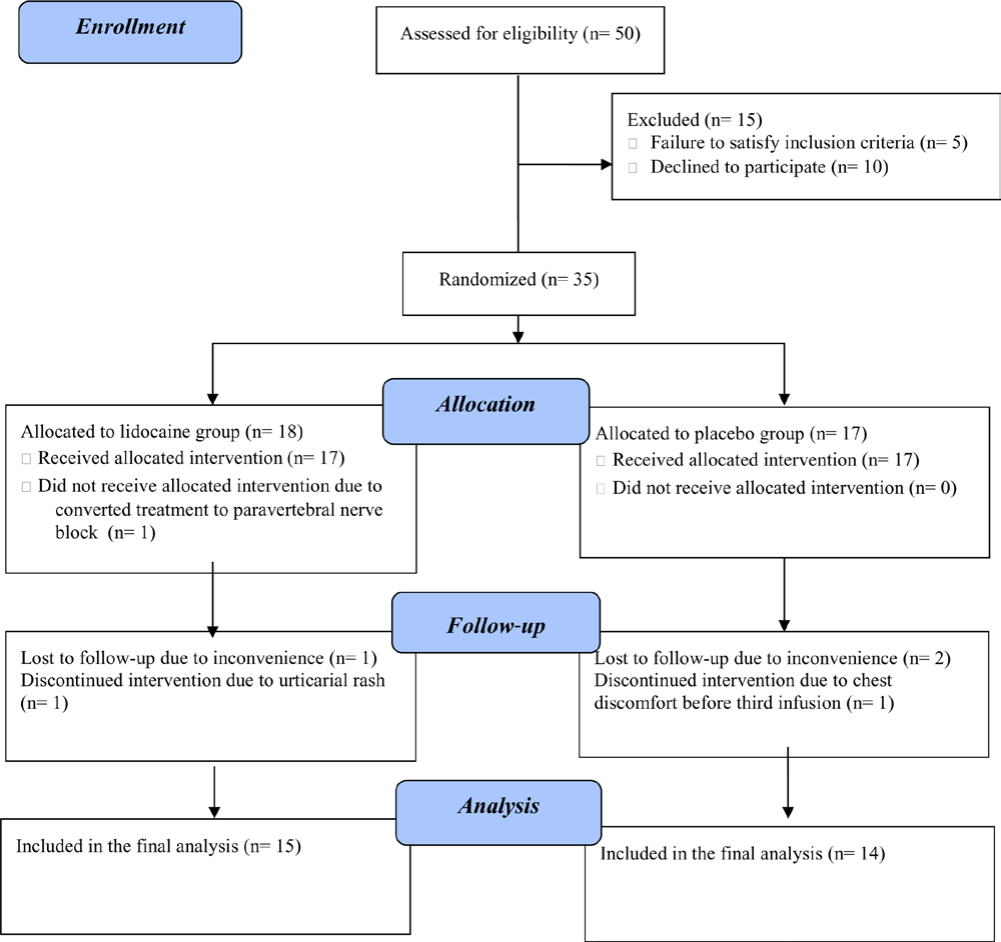
CONSORT flow diagram of the study protocol. About 50 participants were assessed for eligibility before 1:1 randomization into 2 study arms. 35 participants were randomized after excluding 15 participants due to noncompliance with inclusion criteria or declination to participate. Six participants who had withdrawn (n = 3) or were lost to follow-up (n = 3) were excluded after each visit.

Of the 50 individuals with new-onset neuropathic pain recruited for this study, 21 were excluded or withdrawn, primarily because of the inconvenience associated with multiple follow-up visits (Fig. 1). BL demographics were generally comparable between the groups, with no significant differences observed for gender, age, weight, BMI, diagnosis, or BL pain intensity. Most study participants (76%) were diagnosed with peripheral nerve injury (Table 1).

**Table 1:** Demographic and clinical characteristics of neuropathic pain patients.

Characteristics	Lidocaine (n = 15)	Placebo (n = 14)	*P*
Age (yr)	62.0 (30.0–66.5)	51.5 (24.0–68.0)	.793
Sex; male	9 (60%)	9 (64%)	.812
Weight (kg)	64.2 (54.0–71.5)	63.3 (56.1–81.4)	.743
BMI (kg/m^2^)	22.5 (20.3–26.1)	23.8 (23.1–29.4)	.337
Without underlying disease	12 (80%)	7 (50%)	.128
Diagnosis			NA
Peripheral nerve injury	10 (67%)	12 (86%)	
Phantom limb pain	2 (13%)	0 (0%)	
Radicular pain	1 (7%)	1 (7%)	
Postsurgical pain syndrome	1 (7%)	1 (7%)	
Acute herpes zoster	1 (7%)	0 (0%)	
Location of pain			NA
Head	3 (20%)	0 (0%)	
Arm	6 (40%)	6 (43%)	
Leg	2 (13%)	4 (29%)	
Upper body	3 (20%)	1 (7%)	
Lower body	1 (7%)	0 (0%)	
Other	0 (0%)	3 (21%)	
Initial pain intensity			
Maximum pain	7.0 (5.5–8.5)	8.5 (4.0–10.0)	.593
Average pain	6.0 (4.5–7.5)	5.0 (4.0–7.0)	.756
Minimum pain	3.0 (2.0–4.0)	2.5 (1.0–5.0)	.550
DN4 score	5.0 (3.0–8.0)	5.5 (4.0–7.0)	.912

Data presented as median (interquartile range) or n (%). A *P* value < .05 indicates statistical significance using Mann–Whitney *U* test.

BMI = body mass index, DN4 = Douleur Neuropathique 4 questionnaire, NA = not applicable.

Although we planned to investigate and analyze 15 participants per group, the final sample included 14 and 15 participants in the placebo and lidocaine groups. However, the mean difference in posttreatment pain adjusted for pretreatment pain using mean NRS at BL/1^st^ week and age of the participants by analysis of covariance was 0.13 (−1.33 to 1.54). This result indicated that the sample size of the 2 groups was large enough to reach a statistically supported conclusion in pain difference at the 12^th^ week if that difference was clinically meaningful (i.e., a reduction of 2.0 or greater on the 0–10 NRS).

### 3.2. Pain reduction

**Figure 2.**
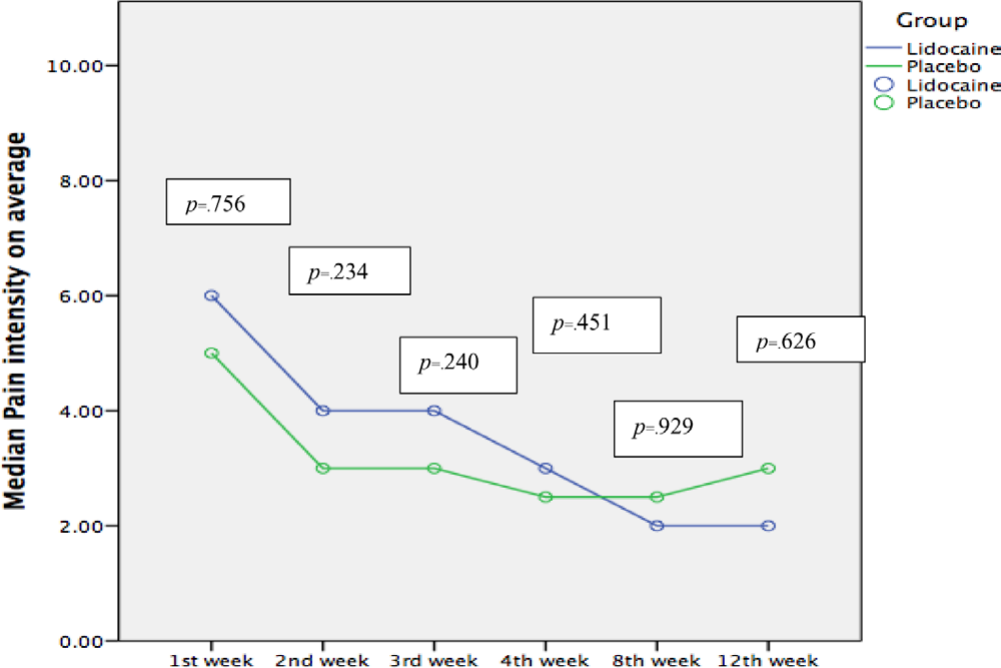
Comparing median average pain intensity over time between Lidocaine and Placebo groups. Data were measured as numeric rating scale (0–10) values and was presented along with corresponding *P* values as average median pain intensity for weeks 1 to 12. * indicates statistical significance (*P* < .05) analyzed using Mann–Whitney *U* test.

**Figure 3.**
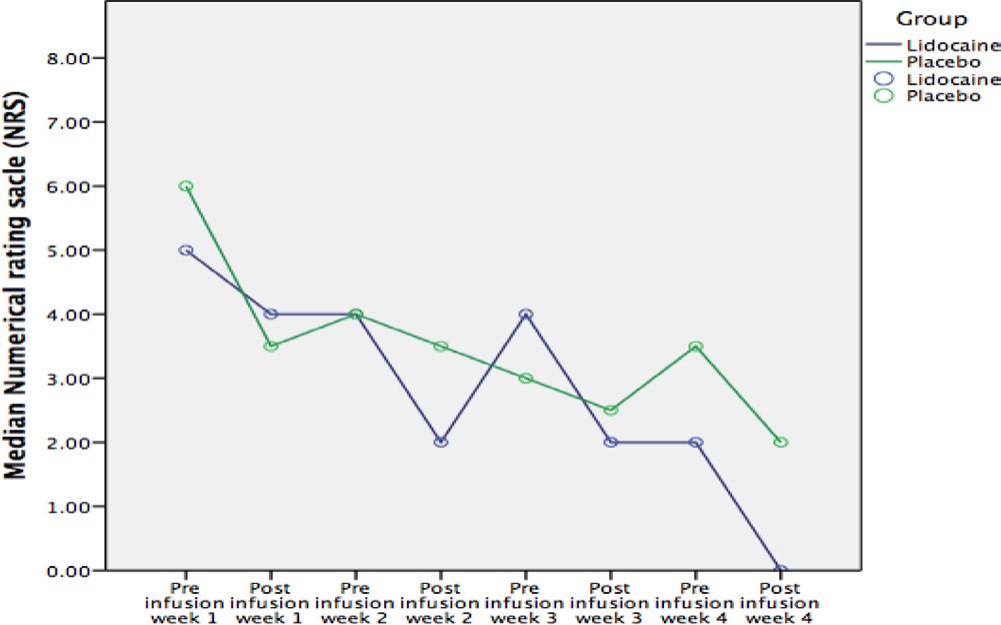
Comparing pain intensity pre- and postinfusion between Lidocaine and Placebo groups. Data was measured and presented as a numeric rating scale (0–10) 15-min pre- and postinfusions in weeks 1 to 4. * indicates a statistically significant difference (*P* < .05) between groups using Mann–Whitney *U* test.

The analyses showed that the median pain intensity from BL to the 12^th^ week decreased significantly for both groups from BL to week 12; 6.0 (4.5–7.5) to 2.0 (1.0–2.5) (*P < *.001) in the lidocaine group, and 5.0 (4.0–7.0) to 3.0 (1.0–4.0) (*P < .*001) in the placebo group. However, there were no statistically significant differences in pain reduction between the 2 groups at any of the assessment points (Fig. 2). The effects sizes (*r*s) calculated with the Mann–Whitney U test at each time point from 1^st^ week, 2^nd^ week, 3^rd^ week, 4^th^ week, 8^th^ week, and 12^th^ week were 0.27, 0.23, 0.22, 0.14, 0.02, and 0.09, respectively. Pain pre- to postinfusion also decreased significantly (*P <* .05) for both groups at all 4 infusion visits. The median percentage of pain reduction was comparable between the 2 groups, and only pain intensity after infusion at week 4 was significantly lower in lidocaine group (*P = *.048) relative to placebo group (Table 2; Fig. 3). After a linear-mixed model was performed, there were no statistically significant differences between the Lidocaine and Placebo groups (fixed factor) in the adjusted average pain intensity with times and tramadol dosage (covariates). The effect estimate (*beta*) was −0.179; 95%CI (−1.754 to 1.397) (*P = *.826). DN4 score was reduced to some extent (but not statistically significant) in both groups (lidocaine: 5.0 [3.0–8.0] to 4.0 [3.5–6.0], *P* = .111; placebo: 5.5 [4.0–7.0] to 4.5 [4.0–7.0], *P* = .323), and was not significantly different between groups (*P* = .626) from BL to week 12. The median total percentage of pain reduction from BL to week 12 was larger in the lidocaine group (62.5 [33.3–81.7]) than in the placebo group (53.6 [25.0–75.0]), but this difference was not statistically significant (*P* = .683).

**Table 2:** Pain intensity pre- and postinfusion and percentage of pain reduction of Lidocaine and Placebo groups.

	Lidocaine (n = 15)	Placebo (n = 14)	*P*
Pain score at first week			
-Preinfusion	5.0 (5.0–6.0)	6.0 (4.0–8.0)	.550
-Postinfusion	4.0 (2.0–5.0)	3.5 (2.0–5.0)	.877
*P*	.003*	.009*	
-% pain reduction	20.0 (6.3–51.4)	26.8 (0.0–44.4)	.707
Pain score at second week			
-Preinfusion	4.0 (3.0–6.0)	4.0 (3.0–6.0)	.860
-Postinfusion	2.0 (0.0–5.0)	3.5 (0.0–4.0)	.654
*P*	.007*	.005*	
-% pain reduction	42.9 (0.0–80.0)	33.3 (0.0–42.9)	.911
Pain score at third week			
-Preinfusion	4.0 (2.0–6.0)	3.0 (2.0–6.0)	.826
-Postinfusion	2.0 (0.0–4.0)	2.5 (0.0–3.0)	.858
*P*	.003*	.007*	
-% pain reduction	25.0 (7.1–38.8)	33.3 (0.0–50.0)	.722
Pain score at fourth week			
-Preinfusion	2.0 (0.0–3.0)	3.5 (0.0–5.0)	.164
-Postinfusion	0.0 (0.0–0.5)	2.0 (0.0–3.0)	.048*
*P*	.048*	.031*	
-% pain reduction	0.0 (0.0–100.0)	10.0 (0.0–33.3)	.780

Data are presented as median (interquartile range).

* *P* < .05 indicated statistical significance using Mann–Whitney *U* test and Wilcoxon Sign Rank test.

### 3.3. Pain medication

The median gabapentin dose was lower in the lidocaine group than in the placebo group at all time points, but this difference was not statistically significant (Table 3). The number and percentage of participants taking tricyclic antidepressants were not significantly different between groups at any time point (*P* range 0.450–0.893). There was, however, a significantly lower median tramadol dose (mg/day) in the lidocaine group from the 9- to 12-week assessment points (0.0 [0.0–68.8] vs 150.0 [0.0–150.0], *P = *.023).

**Table 3:** Daily pain medication taken over 12 wk (weeks 1–12)

Drugs (mg)	Lidocaine (n = 15)	Placebo (n = 14)	*P*
Gabapentin			
1st to 2nd week	600.0 (300.0–1050.0)	450.0 (300.0–900.0)	.690
2nd to 3rd week	600.0 (375.0–1050.0)	750.0 (300.0–1200.0)	.550
3rd to 4th week	600.0 (300.0–1200.0)	900.0 (300.0–1800.0)	.364
5th to 8th week	600.0 (300.0–1200.0)	1050.0 (300.0–1800.0)	.356
9th to 12th week	600.0 (300.0–900.0)	900.0 (200.0–2100.0)	.417
Tramadol			
1st to 2nd week	100.0 (0.0–150.0)	68.8 (50.0–200.0)	.520
2nd to 3rd week	100.0 (0.0–150.0)	150.0 (37.5–200.0)	.282
3rd to 4th week	50.0 (0.0–125.0)	150.0 (37.5–200.0)	.211
5th to 8th week	0.0 (0.0–125.0)	150.0 (37.5–200.0)	.132
9th to 12th week	0.0 (0.0–68.8)	150.0 (0.0–150.0)	.023*

The data are presented as median (interquartile range).

* *P *< .05 indicates statistical significance.

### 3.4. Psychological effects and quality of life

Both groups reported significant improvements in the measures of psychological function and quality of life from BL to the 12-week assessment point (Table 4). In addition, although the percentage of participants who reported moderate to severe depression decreased significantly (*P* = .031) and reported moderate to severe anxiety decreased significantly (*P* = .031) only in the lidocaine group, there were no statistically significant differences between groups in these outcomes. The EQ-5D-5L utility score increased significantly from BL to week 12 in both groups, but there were no significant between-group differences in this outcome at pretreatment (BL) or posttreatment (week 12). The VAS rating of overall perceived health in lidocaine group showed significant improvement from BL to week 12 (*P* = .010), but there were no significant differences between groups at week 12 (posttreatment) (Table 5).

**Table 4:** DASS-21 scores comparison of psychological effects between Lidocaine and Placebo groups: Pretreatment (baseline) versus posttreatment (week 12).

Measurement tools	Lidocaine (n = 15)	Placebo (n = 14)	*P*
Depression			
Pretreatment			.057
- Normal to mild	8 (53.3)	6 (42.9)	
- Moderate to extremely severe	7 (46.7)	8 (57.1)	
Posttreatment			.330
Normal to mild	14 (93.3)	11 (78.6)	
Moderate to extremely severe	1 (6.7)	3 (21.4)	
*P*	*.031* *	.063	
Anxiety			
Pretreatment			.837
Normal to mild	8 (53.3)	8 (57.1)	
Moderate to extremely severe	7 (46.7)	6 (42.9)	
Posttreatment			.169
Normal to mild	14 (93.3)	10 (71.4)	
Moderate to extremely severe	1 (6.7)	4 (28.6)	
*P*	*.031* *	.625	
Stress			
Pretreatment			.588
Normal to mild	9 (60.0)	7 (50.0)	
Moderate to extremely severe	6 (40.0)	7 (50.0)	
Posttreatment			.330
Normal to mild	14 (93.3)	11 (78.6)	
Moderate to extremely severe	1 (6.7)	3 (21.4)	
*P*	.063	.125	

The data are presented as n (%).

DASS-21 = Depression Anxiety Stress Scales-21.

* *P* < .05 indicates statistical significance.

**Table 5:** EQ-5D-5L Utility and VAS scores comparison between Lidocaine and Placebo groups: Pretreatment (baseline) versus posttreatment (week 12).

	Lidocaine (n = 15)	Placebo (n = 14)	*P*
EQ-5D-5L (utility)			
Pretreatment	0.693 (0.568–0.877)	0.709 (0.447–0.877)	.913
Posttreatment	0.877 (0.808–0.943)	0.910 (0.817–1.000)	.552
* P*	.019*	.003*	
EQ-5D-5L (VAS)			
Pretreatment	60.0 (50.0–70.0)	70.0 (60.0–75.0)	.198
Posttreatment	80.0 (72.5–85.0)	80.0 (70.0–90.0)	.840
* P*	.010*	.074	

The data are presented as median (IQR).

EQ-5D-5L = EuroQol Group questionnaire with 5 dimensions and 5 levels of severity, VAS = visual analog scale.

* *P* < .05 indicates statistical significance.

### 3.5. Lidocaine infusion-related adverse effects

Dizziness occurred in 4 participants (27%) in the lidocaine group. One participant in the lidocaine group developed urticaria after the second infusion. We then learned that this individual had consulted a dermatologist for treatment of urticaria before entering the study. The decision was subsequently made to withdraw this participant from the analyses. No serious adverse events were observed in either group.

## 4. Discussion

The study finding is that a serial lidocaine infusion of 3 mg/kg once a week for 4 weeks in participants with recent onset of peripheral nerve pain does not provide meaningful benefits with trivial effects (effect size < 0.10) in terms of pain reduction, quality of life, and psychological outcomes, up to 12 weeks after the initial infusion.

Given that intravenous lidocaine infusion has the potential to provide pain relief by blocking sodium channels, which are thought to be the primary pain-generating mechanisms in individuals with peripheral neuropathic pain, we anticipated that the participants who received lidocaine would report larger immediate and longer-term decreases in pain than participants in the placebo group, including at week 12 (the primary endpoint of the current trial). However, we did not observe any significant between-group differences in pain intensity—including immediate effects after infusion—or in measures of psychological function and quality of life at any time point. The only significant effect that emerged was that the lidocaine group reported significantly less use of tramadol than the placebo group from the ninth to the twelfth week. Overall, the findings suggest that lidocaine infusions are not a particularly effective strategy for treating new-onset neuropathic pain.

### 4.1. Effect of systemic lidocaine and its dosage

Previous studies have reported effective lidocaine doses that ranged from 1 to 5 mg/kg.^[29]^ Zhao et al reported that 3 mg/kg of intravenous lidocaine could suppress spinal neuronal activity induced by noxious electrical stimulation in functional magnetic resonance imaging of the hind paws in a dog model. Based on these findings as a group and with a goal of maximizing efficacy while minimizing adverse events, we selected 3 mg/kg as the lidocaine dose for this study.

However, in 2018, Kim et al^[27]^ reported that infusing 3 mg/kg of lidocaine for 1 hour once a week for 4 weeks in a sample of individuals with chronic neuropathic pain showed a significantly more reduction in pain intensity than in placebo group at the 4^th^ week, but there was no detectable pain reduction difference at the 8-week follow-up assessment. Our study confirmed these findings and extended the results to individuals with early neuropathic pain setting (e.g., <6 months duration in this study). In 2019, a systematic review and meta-analysis conducted by Bo Zhu et al^[16]^ concluded that a single lidocaine infusion ranging from 1 to 7.5 mg/ kg was superior to placebo in relieving neuropathic pain in the early postinfusion period. Still, multiple lidocaine infusions of 3 mg/kg and 240 mg in chronic neuropathic pain and fibromyalgia over a period of once a week for 4 weeks have not been shown to have persistent benefits at 4 weeks and 8 weeks.^[17,27,30,31]^

In 1 previous study, the analgesic duration of 5 mg/kg lidocaine infusion was 6 hours longer than placebo, and some study participants reported a duration of analgesic effect longer than 7 days.^[19]^ Tremont-Lukats et al^[32]^ reported that a 5 mg/kg of lidocaine infusion for 6 hours was more effective than 1 or 3 mg/kg to treat neuropathic pain. Further, Viola et al found that the analgesic duration of a single lidocaine infusion at doses of 5 or 7.5 mg/kg varied between 3 and 28 days in participants with painful diabetic peripheral neuropathy. Therefore, further study of serial lidocaine infusion 5 mg/kg or more should be conducted to test the potential of lidocaine infusions at these doses for longer-term efficacy.

### 4.2. Effect of concomitant pharmacological treatment

The titration of antineuropathic pain drugs, such as anticonvulsants, antidepressants, and weak opioids, may have contributed to reduced pain scores in both groups. Many high-quality neuropathic pain randomized controlled trials have shown that anticonvulsant drugs, such as carbamazepine/oxcarbazepine, gabapentin, pregabalin, and antidepressants (such as amitriptyline) can be efficacious for treating neuropathic pain.^[7]^ Although both groups demonstrated similar results in pain reduction at the end of study period, we also postulated that any improvement in pain outcomes in the treatment group would be accompanied by a reduction in the consumption of pain medications. Consistent with this idea, we found that tramadol use was significantly lower in the lidocaine group from the ninth to the twelfth week after the initial infusion. This finding is also consistent with the findings from a report by Hui Liu et al.^[33]^

### 4.3. Adverse effects of lidocaine infusion

The most common adverse events associated with lidocaine infusion are related to central nervous system, including lightheadedness, dizziness, confusion, lethargy, nausea, vomiting, vision changes, and perioral numbness. Hutson et al^[34]^ reported that adverse effects occurred more often at higher intravenous lidocaine infusion rates, and that a flat-dose trial for intravenous lidocaine administration did not cause serious adverse effects. The systematic review studied the side effects of intravenous lidocaine on neuropathic pain participants and reported that intravenous lidocaine has a low risk of causing adverse effects.^[35]^ We observed that intravenously 3 mg/kg of lidocaine did not elicit any serious adverse events in our study participants.

### 4.4. Limitations

This study has a number of limitations that should be considered when interpreting the findings. First, we did not measure the lidocaine blood plasma level in the study participants, so we were not able to examine the associations between lidocaine level and treatment response. Second, as noted previously, we only evaluated a single lidocaine dose in this study. It is possible that a larger lidocaine dose than that evaluated might result in larger and more long-lasting benefits. Third, this was a pilot study in which the number of analyzed participants was 15 in lidocaine group and 14 in placebo group. Although the number of recruited participants was 50, the withdrawal rate was also high due to the requirement for multiple visits. However, a sample size of 29 was sufficient to provide preliminary estimates of the effects of the intervention on the primary study outcome.

## 5. Summary and conclusions

This pilot study found that although a serial lidocaine infusion of 3 mg/kg once a week for 4 weeks in individuals with recent onset of peripheral neuropathic pain was associated with statistically significant reductions in pain intensity, improved quality of life, and psychological outcomes, these reductions were not significantly larger than those associated with placebo treatment and that the effect was not clinically meaningful (effect size* *< 0.10). However, serial lidocaine infusion at this dose may reduce the use of rescue analgesics. Further research needs to determine if a higher dose of lidocaine (e.g. 5 mg/kg or higher) might result in meaningful and long-lasting pain relief in individuals with early-onset neuropathic pain.

## Acknowledgments

The authors gratefully acknowledge the participants who generously agreed to participate in this study, Ms. Nattaya Bunwatsana and Ms. Sunsanee Mali-ong for general research assistance, and Mr. Suthipol Udompunthurak of the Division of Clinical Epidemiology, Department of Research and Development, Faculty of Medicine Siriraj Hospital, for statistical analysis. We also thank Ms. Janravee Laurujisawat for her nursing care and data recording and Mark Simmerman for professional English proofreading and editing.

## Author contributions

**Conceptualization:** Suratsawadee Wangnamthip, Pramote Euasobhon.

**Data curation:** Suratsawadee Wangnamthip, Kasamabhorn Thiangtham, Mark P. Jensen, Pramote Euasobhon.

**Formal analysis:** Suratsawadee Wangnamthip, Kasamabhorn Thiangtham, Sukunya Jirachaipitak, Pranee Rushatamukayanunt, Mark P. Jensen, Pramote Euasobhon.

**Funding acquisition:** Pramote Euasobhon.

**Investigation:** Suratsawadee Wangnamthip, Kasamabhorn Thiangtham, Sukunya Jirachaipitak, Pranee Rushatamukayanunt, Pramote Euasobhon.

**Methodology:** Suratsawadee Wangnamthip, Sukunya Jirachaipitak, Pranee Rushatamukayanunt, Pramote Euasobhon.

**Project administration:** Suratsawadee Wangnamthip, Pramote Euasobhon.

**Resources:** Suratsawadee Wangnamthip, Sukunya Jirachaipitak, Pranee Rushatamukayanunt, Pramote Euasobhon.

**Software:** Suratsawadee Wangnamthip.

**Supervision:** Suratsawadee Wangnamthip, Mark P. Jensen, Pramote Euasobhon.

**Validation:** Suratsawadee Wangnamthip, Mark P. Jensen, Pramote Euasobhon.

**Visualization:** Suratsawadee Wangnamthip, Kasamabhorn Thiangtham, Sukunya Jirachaipitak, Pranee Rushatamukayanunt, Mark P. Jensen, Pramote Euasobhon.

**Writing – original draft:** Suratsawadee Wangnamthip, Kasamabhorn Thiangtham, Mark P. Jensen, Pramote Euasobhon.

**Writing – review & editing:** Suratsawadee Wangnamthip, Sukunya Jirachaipitak, Pranee Rushatamukayanunt, Mark P. Jensen, Pramote Euasobhon.
